# Notes and News

**Published:** 1905-06-17

**Authors:** 


### NOTES AND NEWS.

An epidemic of typhoid has occurred at Bridgend, in Wales.

On Wednesday the new premises of the Samaritan Free Hospital for Women were opened.

A Commission has been appointed by the Paris Academy of Medicine to consider whether it is not advisable to prohibit the application of Röntgen rays by unqualified persons.

The home visiting of patients in connection with the Leeds Public Dispensary is a very important part of the work of the institution. Recently a fourth district has been added to the area already served. Three resident medical officers have been engaged in visiting hitherto, and last year 3,352 patients were visited in their own homes.

The enormous death-rate in India year by year is attracting attention in Parliament. In 1904 the deaths amounted to 1,022,299. It is difficult for people in this country to realise the stubborn barrier of prejudice which the native population shelter behind as a reason for resisting the most obvious sanitary precautions and measures.

A new medical journal entitled *The Transvaal Medical Journal* will appear about August 1, 1905, and will contain among its features an epitome of all current medical literature. This will be abstracted from the publications of all countries, and classified under the headings of Medicine, Surgery, Gynæcology and Obstetrics, Children's Diseases, Tropical Medicine, Public Health, Pathology and Bacteriology, Forensic Medicine, Diseases of the Eye, Diseases of the Ear, Nose and Throat, Skin and Venereal Diseases, etc., by representative men of each of these branches. It will be published under the auspices of the Transvaal Medical Society.

The annual dinner of King's College will be held on Monday, June 26th, at the Hotel Cecil, Colonel R. Jelf presiding.

A strike amongst public vaccinators has occurred in Lower Austria, and in a particular district it is reported that for the past three months no child has been vaccinated.

Princess Louise Augusta of Schleswig-Holstein will open a garden fête and bazaar for the Homes for Working Boys at Sir George Newnes' grounds, "Wildcroft," Putney Heath, at 3 p.m. on June 30th.

A Café Chantant, under the patronage of the Princess Louise, Duchess of Argyll, will take place at the Hôtel Cecil on Wednesday, July 5th, on behalf of the Regimental Homes and Benefits Agency, of which her Royal Highness is president.

The University of Manchester and the Royal Infirmary are about to collaborate in their pathological departments, and the Professor of Pathology at the University will become an honorary member of the medical staff of the infirmary. The arrangement may be only a temporary one, which may lapse on the completion of the new infirmary.

The Medical Officer of Health for Gorton expresses unsatisfactory opinions of hospitals as a means of checking the spread of scarlet fever. He says, "If patients be kept at home the germs die out gradually in the fresh air, but in the hospital they are in the midst of dozens of other cases in all stages of the disease. On their return to school these germs are called into activity by the foul air in the school, and the disease is spread to others by coughing," etc.

A Birmingham University Club is being formed. An excellent club-house joining the University Buildings has been built at a cost of £6,500, and this it is hoped will be ready for use in October. The accommodation is very complete. The council of the University and some generous friends have provided the sum required for building purposes, but £1,500 is still required for furnishing. Towards this a number of the members of the University, and some of the students have already contributed.

Mr. J. D. Cobbold has offered to build an annexe to the Suffolk Convalescent Home at Felixstowe for the treatment of tuberculous children, at a cost of £2,000, and he also offers to give £50 annually towards maintenance. It has not yet been decided to accept the offer, which will entail a considerably enlarged expenditure, and introduce somewhat the character of a hospital into the work of the institution which is now occupied in treating those already convalescent, the care of whom is of course less expensive than that of children who may call for much surgical attention, and are liable to acute illness.

